# Supplementary material for: Targeted Destruction of S100A4 Inhibits Metastasis of Triple Negative Breast Cancer Cells
Source: Biomolecules. 2023 Jul 10;13(7):1099. doi: 10.3390/biom13071099 (PMC10377353; doi:10.3390/biom13071099)
Supplement: Supplementary file 1 [file biomolecules-13-01099-s001.zip › biomolecules-2396048-supplementary.pdf]

Supplementary Methods, Tables and Figures

Supplementary Methods

Chemical Synthesis

Synthesis of US-10113 (Supplementary Fig. S1), RGC and iRGC is summarised in Supplementary Figure S2. US-10113 and intermediate compounds **3b** and **3c** are synthesised using methodology reported by Li et al. (2015) [39] by refluxing compounds **1a-b** with **2a-b** in acetic acid. Compound **3b** was then reacted with pentafluorophenol to incorporate a better leaving group to form pentafluoro compound **5a**, prior to reaction with aminobutylacetamide(ABA)-linked thalidomide compound **13**. Compound **3c** was reacted directly with ABA-linked thalidomide compound **13** in the coupling step described below. The ABA-linked thalidomide compound **13** was synthesised using adapted methodology from Fischer et al. (2014) [40] and Winter et al. (2015) [32]. Finally, the amide bond formation between compounds **5a/3c** and **13** was achieved to yield the bifunctional active endo PROTAC RGC-01-05-18 (**14a**), termed RGC for short, and inactive exo isomer iRGC (**14b**).

General Experimental Details

All chemicals and solvents were purchased from Sigma Aldrich, Fluorochem, Alfa Aesar, Fischer, and Acros Organics with no further purification required before use. Anhydrous DMF and DCM was obtained from an MBRAUN MBSPS5 solvent purification system. TLC was carried out on Merck silica gel 60 F-254 aluminium backed plates. Compounds were observed *via* exposure to UV light or treatment with appropriate staining solutions such as potassium permanganate or ninhydrin, and then developed *via* a heat gun. Column chromatography was performed using silica (40-63 µM) supplied by Sigma Aldrich. All columns used had the silica made into a slurry with the appropriate solvent system to provide the column mobile phase and layered

onto sand. The product was absorbed onto silica and loaded onto the top of the mobile phase. Column was then eluted using the desired eluent system and the desired fractions were collected. Compounds were characterised by ^{1}H (400 MHz) and ^{13}C (101MHz) NMR spectra were recorded on a Bruker AMX400 spectrometer (^{1}H 400 MHz; ^{13}C 101 MHz) in the required deuterated solvent. Deuterated solvents were obtained from Sigma Aldrich with an internal standard tetramethyl silane (TMS). Chemical shifts are reported as ppm (parts per million) and reported from downfield to upfield. Coupling constant (*J*) are reported in Hz. Splitting reported as singlet – s, broad singlet – br s, multiplet – m, d – doublet, t – triplet, q – quartet, s – septet, dd – doublet of doublets, ddd – doublet of doublet of doublets. Low Resolution Mass Spectrometry (LRMS) and High-Resolution Mass Spectrometry (HRMS) were recorded using the analytical service within the Chemistry Department at the University of Liverpool. LRMS and HRMS were conducted on a VG analytical 7070E machine, Frisons TRIO mass spectrometers or Agilent QTOF 7200 using chemical ionisation (CI) or electrospray (ES). Infra-red spectra were recorded in the range of 4000-500 cm^{-1} using a JASCO FT/IR 4200 spectrometer, or Bruker ALPHA FT-IR platinum ATR spectrometer. Melting points (MP) were carried out using a Gallenkamp melting point machine and values were recorded in degrees Celsius with three concordant results. HPLC was carried out using Agilent 1200 HPLC equipped with a ZORBAX Eclipse Plus C18 column (4.6mm x 10 mm, 3.5 Pm) at 25 qC. Flow rate 0.5 mL/ min for 15 minutes using MeOH/ Water with compounds dissolved in methanol. UV detector recorded signals at 254 nm. HPLC Method: min, gradient: 80% H_2O hold to 2 min, 80-5% H2O in MeOH to 12 min, then hold at 80% H_2O to 15 min.

General Procedures

General Procedure A

To a screw fix sealed tube, (3a*R*,4*R*,7*S*,7a*S*)-Tetrahydro-4,7-methanoisobenzofuran-1,3-dione derivative (1.6 equiv.) (**1a-b**) and a functionalised aniline (**2a-b**) (1 equiv.) were suspended in glacial acetic acid (1.2 M) and allowed to stir overnight at reflux. After this time, the solvent was concentrated under reduced pressure. The resultant solid was dissolved in EtOAc and washed with distilled water (x2) and brine (x1). The organic phase was dried over $MgSO_4$, filtered, and concentrated *in vacuo* to yield a

crude solid. Purification *via* column chromatography eluting with 0-50% EtOAc in *n*-hexane afforded the desired product.

Synthesis of (3a*R*,4*S*,7*R*,7a*S*)-2-(*o*-Tolyl)-3a,4,7,7a-tetrahydro-1*H*-4,7-methanoisoindole-1,3(2*H*)-dione (3a, US-10113)

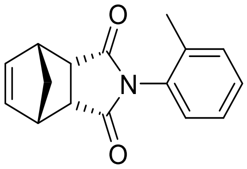

Reaction repeated as General Procedure A using *o*-toluidine (**2a**) gave the title compound (**US10113** (**3a**)) as a white solid (0.64 g, 54%). ^{1}H NMR (400 MHz, DMSO-*d*6) δ 7.37 (m, 2H, Ar-H), 7.30 (m, 1H, Ar-H), 7.13 (dd, 1H, *J*= 7.7, 16.2 Hz, Ar-H), 6.38 (m, 2H, C=C-H), 3.23 (m, 2H, ((CH)$_2$C=O)C-H), 2.95 (m, 2H, ((CH)$_2$CH$_2$)C-H), 2.09 (d, 3H, *J*= 8.0 Hz, CH$_3$), 1.56 – 1.47 (m, 2H, CH$_2$). ^{13}C NMR (101 MHz, DMSO-*d*6) δ 177.2, 138.2, 135.7, 132.3, 131.2, 129.5, 129.1, 128.4, 127.8, 48.6, 48.0, 45.5, 44.9, 43.5, 17.6. IR ν_{max}/cm^{-1} (solid) 2976 (m), 1774 (s), 1376 (s), 1194 (s), 717 (s). HRMS (CI+) m/z calculated for $C_{16}H_{16}NO_2$: 254.1181. Found [M+H]$^+$: 254.1183 (Diff 0.79 ppm). MP: 142–143°C. Purity HPLC 96.6%, R_t= 2.1 min.

Synthesis of 4-((3a*R*,4*S*,7*R*,7a*S*)-1,3-Dioxo-1,3,3a,4,7,7a-hexahydro-2*H*-4,7-methanoisoindol-2-yl)-3-methylbenzoic acid (3b)

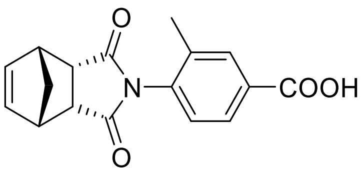

Reaction repeated as General Procedure A using (3a*R*,4*S*,7*R*,7a*S*)-3a,4,7,7a-tetrahydro-4,7-methanoisobenzofuran-1,3-dione (**1a**) and 4-amino-3-methylbenzoic acid (**2b**) gave the title compound (**3b**) as a pale-pink solid (1.66 g, 84%). ^{1}H NMR (400 MHz, CD$_3$OD-*d*4) δ 7.96 (d, 1H, *J*= 8.0 Hz, Ar-H), 7.89 (d, 1H, *J*= 8.0 Hz, Ar-H), 7.21 (d, 0.5H, *J*= 8.0, Ar-H), 7.02 (d, 0.5H, *J*= 8.0 Hz, Ar-H), 6.32 (m, 2H, C=C-H), 3.60 (m, 2H, ((CH)$_2$C=O)C-H), 3.42 (m, 2H, ((CH)$_2$CH$_2$)C-H), 2.15 (s, 3H, CH$_3$), 1.72-1.69

(m, 2H, CH_2). ^{13}C NMR (101 MHz, CD_3OD-*d*4) δ 179.3, 169.6, 139.8, 139.6, 138.5, 133.9, 133.7, 130.5, 129.8, 129.8, 50.5, 47.6, 47.0, 44.9, 44.5, 19.3. HRMS (ES+) *m/z* calculated for $C_{17}H_{16}NO_4$: 298.1079. Found $[M+H]^+$: 298.1084 (Diff 1.68 ppm).

Synthesis of 4-((3a*R*,4*R*,7*S*,7a*R*)-1,3-Dioxo-1,3,3a,4,7,7a-hexahydro-2*H*-4,7-methanoisoindol-2-yl)-3-methylbenzoic acid (3c)

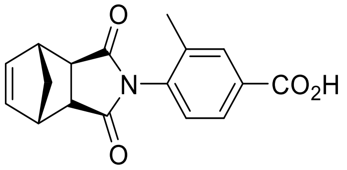

Reaction repeated as General Procedure A using (3a*R*,4*R*,7*S*,7a*R*)-3a,4,7,7a-Tetrahydro-4,7-methanoisobenzofuran-1,3-dione (**1b**) and 4-amino-3-methylbenzoic acid (**2b**) gave the title compound (**3c**) as a pale-pink solid (1.84 g, 93%). ^{1}H NMR (400 MHz, CD_3OD-*d*4) δ 7.91 (d, 1H, *J*= 4.0 Hz, Ar-H), 7.84 (d, 1H, *J*= 12.0 Hz, Ar-H), 7.11 (d, 0.5H, *J*= 8.0 Hz, Ar-H), 7.06 (d, 0.5H, *J*= 8.0 Hz, Ar-H), 6.30-6.29 (m, 2H, C=C-H), 3.25-3.22 (m, 2H, ((CH)$_2$C=O)C-H), 2.86-2.83 (m, 2H, ((CH)$_2$CH$_2$)C-H), 2.11 (s, 3H, CH_3), 1.56-1.47 (m, 2H, CH_2). ^{13}C NMR (101 MHz, CD_3OD-*d*4) δ 179.3, 179.3, 169.6, 139.8, 139.6, 133.9, 133.8, 130.5, 129.8, 129.4, 50.5, 47.7, 47.0, 19.3. HRMS (CI+) *m/z* calculated for $C_{17}H_{16}NO_4$: 298.1079. Found $[M-H]^+$: 298.1082 (Diff 1.01 ppm).

Synthesis of Perfluorophenyl 4-((3a*R*,4*S*,7*R*,7a*S*)-1,3-dioxo-1,3,3a,4,7,7a-hexahydro-2*H*-4,7-methanoisoindol-2-yl)-3-methylbenzoate (5a)

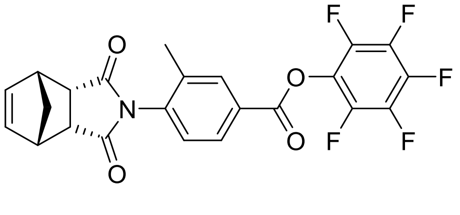

To a flask, carboxylic acid-derivative (**3b**) (1 equiv.), 2,3,4,5,6-pentafluorophenol (**4**) (1 equiv.), DMAP (0.1 equiv.), and EDC.HCl (1 equiv.) were suspended in anhydrous THF (0.2 M) and allowed to stir at room temperature overnight under a nitrogen atmosphere. After this time, the crude mixture was filtered, and the filtrate was concentrated under reduced pressure. Purification *via* column chromatography eluting with 0-40% EtOAc in *n*-hexane afforded the title compound (**5a**) as a yellow solid (42.2 mg, 54%). ^{1}H NMR (400 MHz, CD_3OD-*d*4) δ 8.06 (d, 1H, *J*= 8.0 Hz, Ar-H), 7.98 (d, 1H, *J*= 8.0 Hz, Ar-H), 7.23 (d, 0.5H, *J*= 8.0 Hz, Ar-H), 7.04 (d, 0.5H, *J*= 8.0 Hz, Ar-H), 6.23 (m, 2H, C=C-H), 3.52-3.50 (m , 2H, $((CH)_2C{=}O)C$-H), 3.22-3.20 (m, 2H, $((CH)_2CH_2)C$-H), 2.13-2.09 (d, 3H, *J*= 16.0 Hz, CH_3), 1.71-1.58 (m, 2H, CH_2). ^{13}C NMR (101 MHz, CD_3OD-*d*4) δ 177.2, 165.3, 146.0, 143.7, 141.1, 140.9, 139.5, 137.4, 133.6, 130.2, 126.9, 120.9, 53.4, 46.1, 45.7, 18.1. HRMS (ES+) *m/z* calculated for $C_{23}H_{15}{}^{19}F_5NO_4$: 464.0921. Found $[M+H]^+$: 464.0932 (Diff 2.37 ppm).

Synthesis of *Tert*-Butyl (4-aminobutyl)carbamate (7)

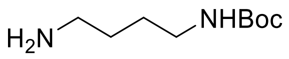

A solution of Boc_2O (1.24 g, 5.68 mmol, 0.1 equiv.) in $CHCl_3$ (30 mL, 0.9 M) was slowly added *via* a dropping funnel to a flask charged with 1,4-diaminobutane (**6**) (5 g, 26.6 mmol, 1 equiv.) and $CHCl_3$ (60 mL, 0.4 M), at a rate of 1 drop per 5 seconds. The solution was allowed to stir at room temperature for 16 hours. After this time, the crude mixture was washed with saturated aq. $NaHCO_3$ solution (x1), water (x1), and brine (x1). The organic phase was dried over $MgSO_4$, filtered, and concentration under reduced pressure to yield a crude brown oil. Purification *via* column chromatography eluting with 10-20% EtOAc in *n*-hexane yielded the title compound (**8**) as a yellow oil (9.39 g, 88%). ^{1}H NMR (400 MHz, CD_3OD-*d*4) δ 7.89 (br s, 1H, N-H), 3.03 (t, 2H, *J*= 8.0, 12.0 Hz, CH_2), 2.62 (t, 2H, *J*= 8.0, 16.0 Hz, CH_2), 1.48-1.45 (m, 6H, $(CH_2)_2$), NH_2), 1.42 (s, 9H, $(CH_3)_3$). ^{13}C NMR (101 MHz, CD_3OD-*d*4) δ 149.6, 80.1, 42.0, 35.2, 29.0, 28.7, 28.6, 28.4, 26.3. HRMS (CI+) *m/z* calculated for $C_9H_{21}N_2O_2$: 189.1603. Found $[M+H]^+$: 189.1602 (Diff -0.53 ppm).

Synthesis of *Tert*-Butyl (4-(2-chloroacetamido)butyl)carbamate (9)

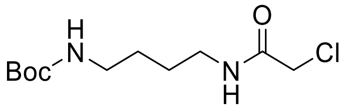

Tert-butyl (4-aminobutyl) carbamate (**7**) (5 mL, 26.6 mmol, 1 equiv.) was dissolved in anhydrous THF (27 mL, 1 M) and cooled to 0°C. 2-Chloroacetyl chloride (**8**) (2.3 mL, 29.3 mmol, 1.1 equiv.) and DIPEA (4.4 mL, 26.6 mmol, 1 equiv.) were added, and allowed to stir at room temperature for 3 hours under a nitrogen atmosphere. After this time, the solution was concentrated under reduced pressure. The residue was diluted with ethyl acetate and washed with water (x3), and brine (x1). The organic phase was dried over $MgSO_4$, filtered, and concentrated *in vacuo* to yield the title compound (**9**) as a brown oil (6.12 g, 87%). ^{1}H NMR (400 MHz, $CDCl_3$) δ 6.66 (br s, 1H, N-H), 4.58 (br s, 1H, N-H), 4.05 (s, 2H,CH_2), 3.33 (dd, 2H, *J*= 12.9, 6.7 Hz, CH_2), 3.16 (m, 2H, CH_2), 1.57 (ddd, 4H, *J*= 9.7, 7.3, 3.9 Hz, $(CH_2)_2$), 1.44 (s, 9H, $(CH_3)_3$). ^{13}C NMR (101 MHz, $CDCl_3$) δ 165.9, 156.0, 79.3, 42.7, 39.5, 28.4, 27.5, 26.6. HRMS (ES+) *m/z* calculated for $C_{11}H_{21}{}^{35}ClN_2O_3Na$: 287.1141. Found $[M+Na]^+$: 287.1133 (Diff -2.79 ppm).

Synthesis of *Tert*-Butyl (4-(2-((1,3-dioxo-1,3-dihydroisobenzofuran-4-yl)oxy)acetamido)butyl)carbamate (11)

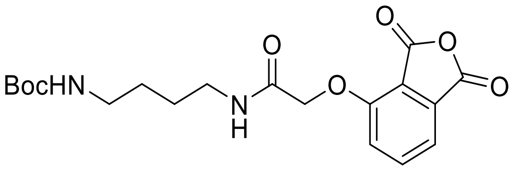

A solution of 4-hydroxyisobenzofuran-1,3-dione (**10**) (1.0 g, 6.09 mmol, 1 equiv.) in anhydrous THF (8.7 mL, 0.7 M) was treated with crude *tert*-butyl (4-(2-chloroacetamido)butyl)carbamate (**9**) (2.42 g, 9.14 mmol, 1 equiv.) and cooled to 0°C. NaH (0.31 g, 7.92 mmol, 1.3 equiv.) was added portion wise and allowed to stir at reflux overnight under a nitrogen atmosphere. After this time, the reaction was diluted with DCM, and washed with water (x1), saturated aq. $NaHCO_3$ solution (x1), and brine (x1). The organic phase was dried over $MgSO_4$, filtered, and concentrated under

reduced pressure to yield a crude orange oil. Purification *via* column chromatography eluting with 0-2% MeOH in DCM yielded the title compound (**11**) as a pale-yellow oil (2.08 g, 72%). ^{1}H NMR (400 MHz, $CDCl_3$) δ 7.64 (dd, 1H, *J*= 7.8, 0.6 Hz, Ar-H), 7.48 (t, 1H, *J* = 8.1 Hz, Ar-H), 7.14 (d, 1H, *J*= 8.1 Hz, Ar-H), 7.08 (br s, 1H, NH), 4.67 (br s, 1H, NH), 4.60 (s, 2H, CH_2), 3.35 – 3.29 (m, 2H, CH_2), 3.12 (d, 2H, *J* = 6.1 Hz, CH_2), 1.56 (m, 4H, 2(CH_2), 1.45 (s, 9H, $(CH_3)_3$). ^{13}C NMR (101 MHz, $CDCl_3$) δ 168.1, 167.5, 165.6, 156.0, 154.5, 131.1, 129.9, 124.9, 123.3, 116.9, 79.1, 68.1, 52.99, 39.5, 38.8, 28.4, 27.2, 26.6. HRMS (ES+) *m/z* calculated for $C_{19}H_{25}N_2O_7$: 393.1662. Found $[M+H]^+$: 393.1674 (Diff 3.05 ppm).

Synthesis of *N*-(4-Aminobutyl)-2-((2-(2,6-dioxopiperidin-3-yl)-1,3-dioxoisoindolin-4-yl)oxy)acetamide (13)

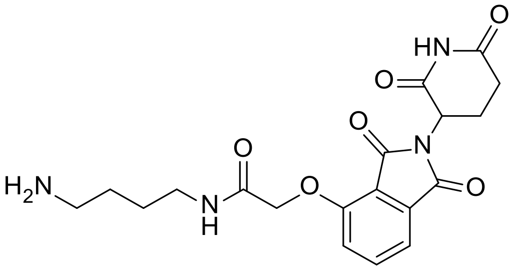

Tert-butyl (4-(2-((1,3-dioxo-1,3-dihydroisobenzofuran-4-yl)oxy)acetamido)butyl)carbamate (**11**) (0.1 g, 0.26 mmol, 1 equiv.), 3-aminopiperidine-2,6-dione (**12**) (0.04 g, 0.29 mmol, 1.1 equiv.) and KOAc (0.06 g, 0.65 mmol, 2.5 equiv.) were dissolved in AcOH (2.6 mL, 0.1 M) and heated in a microwave at 150°C for 12 minutes. After this time, the reaction mixture was cooled and concentrated under air flow to yield a crude dark blue oil. Purification *via* column chromatography with normal phase silica gel eluting with the solvent system 1% water, 2% methanol and 7% ethyl acetate yielded the title compound (**13**) as a yellow oil (28 mg, 28%). ^{1}H NMR (400 MHz, CD_3OD-*d*4) δ 7.57-7.22 (m, 3H, Ar-H), 4.68 (m, 1H, NC-H), 3.78 (s, 2H, CH_2), 2.94 (m, 2H, CH_2), 2.54 (m, 2H, CH_2), 2.49-2.25 (m, 4H, $(CH_2)_2$), 1.64-1.62 (m, 4H, $(CH_2)_2$). ^{13}C NMR (101 MHz, CD_3OD-*d*4) δ 175.4, 170.3, 169.5, 168.0, 167.8, 160.2, 146.3, 134.1, 133.7, 120.6, 115.0, 68.0, 64.0, 44.2, 38.0, 30.7, 29.4, 29.1, 23.0. LRMS (ES+) *m/z* calculated for $C_{19}H_{23}N_4O_6$: 403.2. Found $[M+H]^+$: 403.2.

Synthesis of 4-((3a*R*,4*S*,7*R*,7a*S*)-1,3-Dioxo-1,3,3a,4,7,7a-hexahydro-2*H*-4,7-methanoisoindol-2-yl)-*N*-(4-(2-((2-(2,6-dioxopiperidin-3-yl)-1,3-dioxoisoindolin-4-yl)oxy)acetamido)butyl)-3-methylbenzamide (14a, RGC-01-05-18)

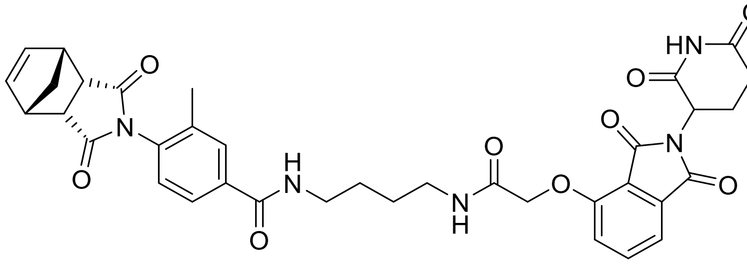

To a flask, perfluorophenyl 4-((3a*R*,4*R*,7*S*,7a*S*)-1,3-hydro-2*H*-4,7-methanoisoindol-2-yl)benzoate derivative (**5a**) (1 equiv.), *N*-(4-aminobutyl)-2-((2-(2,6-dioxopiperidin-3-yl)-1,3-dioxoisoindolin-4-yl)oxy)acetamide (**13**) (1 equiv.), and DMAP (2 equiv.) were suspended in anhydrous DMF (0.2 M) and allowed to stir at 50˚C for 16 hours under a nitrogen atmosphere. After this time, the solution was cooled and concentrated under reduced pressure to yield a crude brown oil. Purification *via* column chromatography using normal phase silica gel eluting with 1% water: 2% methanol: 7% EtOAc), followed by trituration with acetone. If necessary, further purification *via* SCX-2 cartridge eluting with 1M NH_3 in MeOH afforded the title compound (**14a**) as a yellow oil (25 mg, 34%). ^{1}H NMR (400 MHz, CD_3OD-*d4*) δ 8.51-8.47 (m, 2H, Ar-H), 8.21-8.19 (m, 2H, Ar-H), 7.88 (d, 1H, *J*= 8.0 Hz, Ar-H), 7.32-7.25 (m, 1H, Ar-H), 6.29 (m, 2H, C=C-H), 4.71 (s, 2H, CH_2) 4.66 (m, 1H, C-H), 3.78-3.77 (m, 2H, $((CH)_2C{=}O)$C-H), 3.62-3.60 (m, 2H, $((CH)_2CH_2)$C-H), 3.37 (m, 2H, CH_2), 3.21-2.13 (m, 6H, $(CH_2)_2$, CH_2), 2.89 (s, 3H, CH_3), 2.05-1.98 (m, 4H, $(CH_2)_2$), 1.59-1.51 (m, 2H, CH_2). ^{13}C NMR (101 MHz, CD_3OD-*d4*) δ 176.2, 175.1, 170.3, 169.8, 168.7, 168.4, 159.4, 142.6, 138.1, 134.0, 133.5, 133.2, 132.7, 130.7, 129.6, 128.6, 122.4, 120.0, 118.9, 69.1, 64.4, 54.5, 45.6, 45.1, 40.2, 39.4, 30.2, 27.9, 22.2, 18.3. HRMS (ES+) *m/z* calculated for $C_{36}H_{36}N_5O_9$: 682.2513. Found $[M+H]^+$: 682.2522 (Diff 1.32 ppm). IR ν_{max}/cm^{-1} (oil) 3323 (m), 3095 (m), 2979 (w), 1706 (s) 1698 (s), 1684 (s), 1385 (s), 1212 (s). Purity HPLC 94.9%, R_t= 4.9 min.

Synthesis of 4-((3a*R*,4*R*,7*S*,7a*S*)-1,3-Dioxo-1,3,3a,4,7,7a-hexahydro-2*H*-4,7-methanoisoindol-2-yl)-*N*-(4-(2-((2-(2,6-dioxopiperidin-3-yl)-1,3-dioxoisoindolin-4-yl)oxy)acetamido)butyl)-3-methylbenzamide (14b)

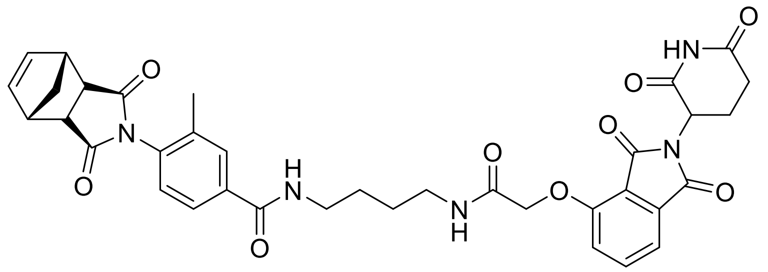

To a flask, *N*-(4-aminobutyl)-2-((2-(2,6-dioxopiperidin-3-yl)-1,3-dioxoisoindolin-4-yl)oxy)acetamide (**13**) (34.5 mg, 0.069 mmol, 1 equiv.) was added and dissolved in DMF (0.35 mL, 0.2 M). The flask was cooled to 0˚C, then HATU (50 mg, 0.14 mmol, 2 equiv.) and DIPEA (0.04 mL, 0.21 mmol, 3 equiv.) were added and contents allowed to stir at room temperature for 10 minutes. 4-((3a*R*,4*R*,7*S*,7a*R*)-1,3-Dioxo-1,3,3a,4,7,7a-hexahydro-2*H*-4,7-methanoisoindol-2-yl)-3-methylbenzoic acid (**3c**) (20 mg, 0.069 mmol, 1 equiv.) was added and contents continued to stir at room temperature for 16 hours. After this time, the solution was concentrated under reduced pressure to yield a crude brown oil. Purification *via* column chromatography using reverse phase silica gel eluting with 5-20% MeCN in H_2O switching to 0-30% MeOH in H_2O, followed by purification *via* a SCX-2 cartridge eluting with 1M NH_3 in MeOH afforded the title compound (**14b, iRGC**) as a yellow oil (18 mg, 24%). ^{1}H NMR (400 MHz, CD_3OD-*d*4) δ 8.55 (m, 1H, Ar-H), 8.16 (t, 1H, *J*= 8.0, 12.0 Hz, Ar-H), 7.81-7.78 (m, 2H, Ar-H), 7.24-7.23 (m, 1H, Ar-H), 7.11-7.10 (m, 1H, Ar-H), 6.22 (m, 2H, C=C-H), 4.67-4.64 (m, 1H, C-H), 4.42 (s, 2H, CH_2), 3.62-3.61 (m, 2H, $((CH)_2C=O)C$-H), 3.51-3.50 (m, 2H, $((CH)_2CH_2)C$-H), 3.31-3.29 (m, 2H, CH_2), 3.24-3.21 (m, 2H, CH_2), 2.76-2.71 (m, 4H, $(CH_2)_2$), 2.13 (s, 3H, CH_3), 1.79-1.76 (m, 2H, CH_2), 1.52-1.49 (m, 4H, $(CH_2)_2$). ^{13}C NMR (101 MHz, CD_3OD-*d*4) δ 176.9, 174.6, 171.6, 171.0, 168.4, 166.3, 160.6, 141.5, 140.5, 134.6, 133.5, 133.0, 133.0, 129.5, 127.6, 125.9, 123.6, 118.0, 117.3, 69.8, 62.9, 55.9, 45.6, 45.2, 40.2, 38.6, 31.2, 28.3, 21.3, 17.9. HRMS (ES+) *m/z* calculated for $C_{36}H_{36}N_5O_9$: 682.2513. Found $[M+H]^+$ 682.2541 (Diff 4.10 ppm). Purity HPLC 94.1%, R_t= 4.2 min. The final compound US-10113 coupled to thalidomide RGC-01-05-18 is referred to as RGC for brevity in the following Methods.

Cytotoxicity assay

For US-10113 on Rama 37 cell line, $2x10^5$ Rama 37 cells in 5 ml of growth medium (GM) (DMEM, 10% (v/v) fetal calf serum (FCS), 10 ng/ml hydrocortisone, 10 ng/ml insulin, 100 units/ml penicillin and 100 mg/ml streptomycin, were plated in 6 plates of 6 cm diameter petri dishes and incubated for 24h at 37˚C in an atmosphere of 10% (v/v) CO_2 and 90% (v/v) air. The medium was replaced by GM containing different concentrations of US-10113 initially dissolved in DMSO which was then diluted to a final concentration of 0.1% (v/v) in GM and incubated for a further 24h. The cells were detached by treatment with trypsin/EDTA and counted using a Coulter Counter. Results are the mean ± SE of three experiments, normalized to that for no US-10113 which is standardised to a value of 100 cells.

For the remainder of the assays, 2000 cells in 100 µl GM were added to each well of a 96-well tissue culture plate and allowed to grow for 24h at 37˚C. Medium was replaced with 100 µl per well of drug-free GM to the blank (first row) and 100 µl per well of GM containing different concentrations of US-10113 (solubilised in DMSO) or RGC (solubilised in water) to the other 7 rows of the plate and incubated for a further 24h. Thereafter medium was removed and cells fixed for at least 1h by adding 100 µl of 10% (w/v) trichloroacetic acid (TCA), washed 5x with deionized water and residual water removed by shaking and inverting on filter paper. Fixed cells were stained with 100µl of Sulforhodamine B (SRB) solution for 30min at room temperature. After removal of SRB, wells were washed 5x with 1% (v/v) TCA. Plates were air-dried by shaking and inverting on filter paper. To solubilize the protein-bound dye, 100 µl of 10 mM unbuffered Tris base was added to the wells, shaken for 10min and the optical density in arbitrary units (A.U.) measured by an automated 96-well plate reader at a wavelength of 564 nm. Results are the mean ± SE of 3 independent experiments of 8 wells for each concentration.

Migration and invasion assays

Transwell migration or invasion assays were carried out [29,33] by adding cells in 100µl GM containing 1% (v/v) fetal calf serum (FCS) to the upper compartments of the

Boyden chambers separated from the lower compartments containing GM with 5% (v/v) FCS by a porous membrane or membrane coated with the model extracellular matrix, Matrigel. The 12 well Boyden chambers contained 6.5mm diameter porous polycarbonate membranes with 8µm pores separating the two chambers (Corning Costar). The media in the upper chambers either contained no compounds or different concentrations of potentially inhibitory compounds. US-10113 was solubilised in DMSO, RGC and iRGC in water, and thalidomide in DMSO; the DMSO was dilute to a final concentration of 0.1% (v/v) in all US-10113/ thalidomide tests including 0 µM. The cultures were incubated for 24h at 37˚C in an atmosphere of 10% (v/v) CO_2, 90% (v/v) air. The top surfaces of the membranes were wiped clean of cells and the lower surfaces stained with Diffquik histochemical kit (Reagena, Toivala, Finland). Cells that had migrated through to the underside of the membrane were counted using a light microscope at 200x magnification. Each concentration of the compound was assayed in 4 wells and the means normalized for the number of cells in the no compound control wells. Each experiment was conducted 3 times (n=3) and the overall mean ± SE is shown as a percentage of cells migrating relative to that with no compound, which was set at 100%. The first experiment (Figure 2) was not normalised and showed absolute number of migrating cells.

Knockdown of S100A4 in TNBC MDA-MB-231 cell line

The transient knockdown experiments were carried out by plating 10^5 cells (70-80% confluent) in fresh GM in 3.5 cm diameter 12 well plates and incubated overnight at 37˚C in an atmosphere of 10% (v/v) CO_2, 90% (v/v) air. The original stock of siRNA to S100A4 (DharmaFECT Transfection Reagents-siRNA cat no L-04792-00-0005) was diluted to 100 µM in 1x siRNA buffer as specified by the manufacturer (Horizon, Cambridge, UK). In separate tubes, 5 µl of 5 µM siRNA were mixed with 95 µl DMEM serum-free medium, while in the other tube, 2 µl of Dharmacon transfection reagent were mixed with 98 µl of DMEM-serum free medium, according to the manufacturer's instructions (Horizon). Both tubes were mixed separately, incubated for 5min at room temperature and then mixed together by gently pipetting and incubated for 20min at room temperature. The GM in the plate was removed from the cells and the siRNA mixture was added to each well, followed by adding 800 µl of GM. The final

concentration of siRNA was 25 nM per well. The plates were incubated at 37˚C in 10% (v/v) CO_2, 90% (v/v) air for 0, 24, 48 and 96h and either isolated for Western blotting with 30 µg of lysate on SDS 15% (w/v) polyacrylamide gels or cells were detached with trypsin/EDTA and 2000 cells per well were assessed for migration in Boydon chambers, after a further 24h.

The permanent transductants were produced by plating 10,000 MDA-MB 231 cells (70-80% confluent) in fresh GM in 6 mm diameter 96 well plates and incubated for 24h at 37˚C in an atmosphere of 10% (v/v) CO_2, 90% (v/v) air. The original SMART vector Lentiviral shRNAs to S100A4 (Cat. no: shRNA-1 V3SH7590-225947421 and shRNA-2 V3SH7590-227036519) to S100P (Cat. no: V3SH7590-225163802) and non-targeted scrambled shRNA were obtained from Horizon (Cambridge, UK). Dilutions were made by mixing separately the lentiviral shRNAs ($1.5x10^8$ TU/ml) for S100A4, S100P, a mixture of both, and non-targeted shRNA all with pre-warmed serum-free DMEM in the presence of 4µg/ml polyprene. The lentiviral mixtures were gently mixed and incubated at room temperature for 30 min. The transduction was performed by replacing the GM in the 96 well plates with 50µl of this lentiviral mixture and allowed to incubate for 6h at 37˚C, then 75µl of GM without antibiotics but containing 10%(v/v) FCS was added to each well and incubated for a further 24h at 37˚C. This Transduction Medium was replaced with Selection Medium (GM containing 2.5µg/ml puromycine) for three days. The cells were then detached by trypsinization, and the culture expanded for freezing. The turboGFP expression was used as a reporter for shRNA expression and consequently gene knockdown, it was determined using a fluorescent microscope (450-500nm) filter. The cell lines produced were S100A4 shRNA-1, S100A4 shRNA-2, S100P shRNA, S100A4/P shRNA, and non-targeted shRNA, respectively. Prior to experimentation, Western blotting of cell lines showed over 95% depletion in S100A4 (S100A4 shRNA-1, shRNA-2), in S100P (S100P shRNA), and in both S100A4 and S100P (S100A4/S100P shRNA) with no change in S100A4 or S100P in non-targeted shRNA. Western blotting and assaying for cell migration were conducted as before, except that 30 µg of cell lysate and 5000 cells per Boyden chamber were loaded, respectively. Experiments were conducted with 3 concentrations of RGC for the full 24h period.

Immunohistochemical staining for S100A4

Histological sections (cut at 4 µm) on Histobond slides were dewaxed in xylene and rehydrated through ethanol to water as previously described [72]. Endogenous peroxidase activity in the tissue sections was blocked by immersing the slides in 100% (v/v) methanol containing 0.05% (v/v) H_20_2 for 20min at room temperature [73]. Rabbit polyclonal anti-S100A4, (Cat:PA5-81468; ThermoFisher Scientific, Runcorn, Cheshire) was applied at a dilution of 1:200 in phosphate buffered saline (PBS), 0.5% (v/v) bovine serum albumen (BSA) pH 7.4 in a moisture chamber at room temperature for 70 min. After thoroughly washing the sections in three changes of PBS, indirect immunohistochemical staining was carried out [74] using a commercially available enhanced horse radish peroxidase (HRP) labelled polymer system, the DAKO EnVision+System, peroxidase (DAB) (Dako Ltd now Agilent Technologies UK, Stockport, Cheshire) [75]. This was prepared according to the manufacturer's instructions. Finally, the sections were washed in running tap water before being counterstained in Mayers' hemalum. They were then dehydrated through graded ethanol and xylene and were mounted in DPX mountant (Merck, Poole, UK).

72. Warburton, M.J.; Mitchell, D.; Ormerod, E.J.; Rudland, P.S. Distribution of myoepithelial cells and basement membrane proteins in the resting, pregnant, lactating and involuting rat mammary gland. *J. Histochem. Cytochem***.** **1982,** *30,* 667-76.

73. Streefkerk, J.G. Inhibition of erythrocyte pseudoperoxidase activity by treatment with hydrogen peroxide following methanol. *J. Histochem. Cytochem.* **1972,** *20,* 829-31.

74. Hsu, S-M.; Raine, L.; Fanger, H. Use of avidin-biotin-peroxide complex (ABC) in immunoperoxide techniques. *J. Histochem. Cytochem.* **1981**, *29,* 577-80.

75. Heras, A.; Roach, C.M.; Key, M.E. Enhanced labelled-polymer system for immunohistochemistry. *XVth Eur Cong Pathol. Copenhagen, Denmark* **1995,** Sept. pp. 3-8.

Supplementary Tables

Supplementary Table S1A. Quantification of Western blots for S100A4 in S100A4-Rama 37 cells with different concentrations of RGC.

Experiment [a] S100A4 (Fig.2C)	Band intensity at different nM RGC [b]							
	0	10	25	50	100	250	500	1000
No 1	0.857	0.223	0.168	0.073	0.000	0.000	0.000	0.000
No 2	0.847	0.221	0.167	0.076	0.000	0.000	0.000	0.000
No 3	0.861	0.224	0.172	0.077	0.000	0.000	0.000	0.000
Mean	0.855	0.223*	0.169*	0.075*	0.000*	0.000*	0.000*	0.000*
SE	0.007	0.001	0.003	0.002	0.000	0.000	0.000	0.000

Supplementary Table S1B. Quantification of Western blots for S100P in S100P-Rama 37 cells with different concentrations of RGC.

Experiment [a] S100P (Fig.2D)	Band intensity at different nM RGC [b]							
	0	10	25	50	100	250	500	1000
No 1	1.366	1.361	1.329	1.438	1.370	1.448	1.462	1.454
No 2	1.384	1.374	1.337	1.442	1.432	1.445	1.444	1.325
No 3	1.398	1.370	1.343	1.438	1.327	1.461	1.485	1.295
Mean	1.382	1.369	1.336	1.440	1.377	1.451	1.464	1.358
SE	0.009	0.004	0.004	0.001	0.031	0.004	0.012	0.049

[a] Individual experiments numbered (No) 1-3, together with their mean and standard error (SE) of (A) Figure 2C or (B) Figure 2D.

[b] Quantification of Western blots of Fig.2C or D; ratio of scanned area under peak band intensity of S100A4 or S100P to that of actin (enhancer used) is recorded (Materials and Methods). *Significantly different from 0 nM (Student's t test, $p<0.05$). All results not normalized.

Supplementary Table S2A. Quantification of Western blots for S100A4 in MDA-MB-23 cells with different concentrations of US-10113.

Experiment [a]	Band intensity at different µM US-10113 [b]							
S100A4 (Fig.3C)	0	5	10	25	50	75	100	200
Exp 1	5.421	5.757	6.14	5.01	5.064	4.977	4.387	0.071
Exp 2	5.043	5.492	6.459	5.136	5.218	4.866	4.504	0.066
Exp 3	5.225	5.615	6.551	5.121	5.098	4.965	4.503	0.073
Mean	5.23	5.621	6.383	5.089	5.127	4.936	4.465	0.070*
SE	0.109	0.076	0.124	0.04	0.047	0.035	0.038	0.002

Supplementary Table S2B. Quantification of Western blots for S100P in MDA-MB-231 cells with different concentrations of US-10113.

Experiment [a]	Band intensity at different µM US-10113 [b]							
S100P (Fig.3C)	0	5	10	25	50	75	100	200
Exp 1	3.402	2.890	3.671	3.427	2.235	2.660	3.439	2.404
Exp 2	3.168	2.783	3.849	3.587	2.310	2.683	3.475	2.375
Exp 3	3.233	2.881	3.891	3.576	2.286	2.581	3.487	2.324
Mean	3.268	2.851	3.804	3.530	2.277	2.641	3.467	2.368*
SE	0.070	0.034	0.067	0.052	0.022	0.031	0.014	0.023

[a] Individual experiments numbered (1-3), together with their mean and standard error (SE) of (A) Figure 3C, S100A4 and (B) Figure 3C, S100P.

[b] Quantification of Western blots of Fig.3C; ratio of scanned area under peak band intensity of S100A4 or S100P to that of actin is recorded (Materials and Methods). * Significantly different from 0 µM (Student's t test, $p<0.05$).

Supplementary Table S3. Quantification of Western blots for S100A4 in MDA-MB-231 cells with different concentrations of RGC.

Experiment [a] S100A4 (Fig.3D)	Band intensity at different nM RGC [b]							
	0	10	25	50	100	250	500	1000
No 1	1.000	0.049	0.023	0.125	0.047	0.042	0.068	0.135
No 2	1.038	0.048	0.028	0.115	0.045	0.049	0.079	0.141
No 3	1.029	0.044	0.036	0.139	0.069	0.064	0.017	0.151
Mean	1.023	0.047*	0.029*	0.126*	0.054*	0.051*	0.055*	0.142*
SE	0.011	0.001	0.004	0.007	0.008	0.007	0.019	0.005

[a] Individual experiments numbered (No) 1-3, together with their mean and standard error (SE) of Figure 3D.

[b] Quantification of Western blots of Figure 3D; ratio of scanned area under peak band intensity of S100A4 to that of actin (enhancer used) is recorded (Materials and Methods). * Significantly different from 0 nM (Student's t test, p<0.05). All results normalised to unity.

Supplementary Table S4A. Quantification of Western blots for S100A4 in MDA-MB-231 cells knocked down by S100A4 siRNA for different times.

Experiment [a] S100A4 (Fig.4A)	Band intensity at different times (hr) [b]			
	0hr	48hr	72hr	96hr
No 1	1.020	0.291	0.142	0.097
No 2	0.990	0.318	0.156	0.093
No 3	1.001	0.277	0.143	0.090
Mean	0.995	0.298*	0.150*	0.093*
SE	0.009	0.012	0.005	0.003

Supplementary Table S4B. Quantification of Western blots for S100A4 in clones of MDA-MB-231 cells knocked down by S100A4 shRNA, S100A4/S100P shRNAs.

Experiment [a] S100A4 (Fig.4C)	Band intensity for different clones with shRNAs [b]			
	Non-targeted shRNA	A4 shRNA-1	A4 shRNA-2	A4/P shRNAs
No 1	0.1804	0.0017	0.0019	0.0117
No 2	0.1954	0.0017	0.0016	0.0120
No 3	0.1883	0.0017	0.0017	0.0117
Mean	0.1881	0.0017*	0.0017*	0.0118*
SE	0.0043	0.0000	0.0001	0.0001

[a] Individual experiments numbered (No) 1-3, together with their mean and standard error (SE) of (A) Figure 4A or (B) Figure 4C.

[b] Quantification of Western blots of Figure 4A/C; ratio of scanned area under peak band intensity of S100A4 to that of actin (enhancer used) is recorded (Materials and Methods). * Significantly different from 0 hr or non-targeted shRNA (Student's t test, $p<0.05$). Results in Table S4A normalised, those in Table S4B not normalised to unity.

Supplementary Table S5. Cell migration details of knockdown of S100A4 in TNBC MDA-MB-231 cells

Transduced cell line[a]	Cell migration for different concentrations of RGC ± SE[b]			
	0 ηM	25 ηM	50 ηM	100 ηM
Non-target shRNA	690±22	209±20[d]	88.7±9.0[d]	48.0±6.6[d]
S100A4 shRNA-1	172±12[c]	135±37	123±1.5	126±4.4
S100A4 shRNA-2	72.7±6.2[c]	63.7±3.4	61.7±1.2	60.0±2.0
S100P shRNA	210±3.5[c]	66.7±7.0[d]	66.0±3.5[d]	62.3±4.7[d]
A4/S100P shRNA	13.3±5.6[c]	11.3±2.4	13.7±1.9	10.0±0.6

[a] Permanently infected MDA-MB-231 cells with short-hairpin (sh) RNAs depleted for S100A4 and/or S100P yielding control non-target shRNA, S100A4 shRNA-1, S100A4 shRNA-2, S100A4/S100P transduced cell lines (Details in Figure 4 and Supplementary Methods).

[b] Number of cells migrating through a semi-permeable membrane in 24h, mean ± SE of 3 independent experiments.

[c] Significantly different from control non-target shRNA cells (Student's t test, $p \leq 0.\ 004$).

[d] Significantly different from RGC (Student's t test, $p \leq 0.003$).

Supplementary Table S6. Effect of RGC on subcellular location of S100A4 in different immunohistochemically stained tissues

RGC[a] mg/kg	Immunostained cells with stained nuclei for S100A4[b]		
	Primary tumour Percent ±SD	Spleen Percent ±SD	Colon Percent ±SD
-	16.3±5.5[c,d]	15.7±1.5[c,e]	13.8±2.9[c,f]
0.007	53.0±0.9[d]	14.6±0.5[e]	15.6±3.4[f]
0.07	51.2±1.7[d]	13.4±2.6[e]	12.4±0.5[f]
0.7	61.7±8.5[d]	14.1±3.4[e]	12.5±1.1[f]
3.5	53.7±1.0[d]	16.7±1.5[e]	12.7±1.2[f]

[a] Mouse 4T1 cells were injected orthotopically and different doses of RGC injected sc on the same day, then every 2 to 3 days into mice until they were sacrificed after 4 weeks, as described in Table 1.

[b] Five randomly selected immunohistochemically stained sections for S100A4 from Table 2 for each dose of RGC injected were scored for positive nuclei and cytoplasm at 400x magnification and the mean percentage positive cells with stained nuclei ± standard deviation (SD) are shown.

[c]Probability of difference between the 3 sets of stained tissues from untreated mice using one-way ANOVA test, F=0.606, 2df, p=0.56. p>0.05 was considered not significant.

[d] Probability of difference between the 5 sets of stained primary tumours from RGC-treated mice using one-way ANOVA test, F=73.56, 4df, $p<0.00001$. $p<0.05$ was considered significant.

[e] Probability of difference between the 5 sets of stained spleens from RGC-treated mice using ANOVA test, F=1.79, 4df, $p=0.17$. $p>0.05$ was considered not significant.

[f] Probability of difference between the 5 sets of stained colons from RGC-treated mice using ANOVA test, F=1.94, 4df, $p=0.14$. $p>0.05$ was considered not significant.

Supplementary Table S7. Effect of inhibitors on preformed metastases in TNBC mouse model *in vivo*

Inhibitor[a]	Dose<br>mg/kg	Metastasis[b] incidence<br>(*p*)[d]	No lung[c] metastases (mean ±SE)<br>(*p*)[e]	No involved[c] organs (mean ±SE)<br>(*p*)[e]	Total no[c]. lesions (mean ±SE)<br>(*p*)[e]
None	-	7 of 7	5.14±1.35	2.86±0.46	8.71±1.97
Thalidomide	2.6	6 of 6<br>(cnc[f])	5.67±3.3<br>(*p*=0.88)	3.00±0.56<br>(*p*=0.84)	9.00±3.66<br>(*p*=0.94)
RGC	0.07	4 of 7<br>(*p*=0.096)	1.57±0.81<br>**(*p*=0.043)**	1.71±0.71<br>(*p*=0.20)	3.86±1.74<br>(*p*=0.088)
RGC	0.7	5 of 6<br>(*p*=0.46)	4.83±1.89<br>(*p*=0.89)	1.67±0.33<br>(*p*=0.67)	5.83±1.96<br>(*p*=0.32)
RGC	3.50	5 of 6<br>(*p*=0.46)	5.33±1.84<br>(*p*=0.93)	1.33±0.33<br>**(*p*=0.022)**	5.33±1.84<br>(*p*=0.24)

[a] Mouse TNBC LT1 cells were injected intravenously (iv) (10,000 cells) into female 4-week-old Balb/c mice and the drug was injected (sc) every 3 days, starting 14 days later and continued for a further 15 days. Mice were sacrificed after 29 days (Materials and Methods).

[b] Metastasis incidence was the number of mice with any experimental metastases/number of successfully injected mice.

[c] Number (No) of lung metastases, number of tumour-involved organs or total number of metastatic lesions was shown as mean per mouse ± SE of 6 or 7 successfully injected mice.

[d] Probability of the difference from no inhibitor was calculated using Fisher's Exact test, 2-sided, $p<0.05$ was considered significant.

[e] Probability of the difference from no inhibitor was calculated using Student's t-test, 2-sided, $p<0.05$ was considered significant. [f] Cannot compute.

Supplementary Figures

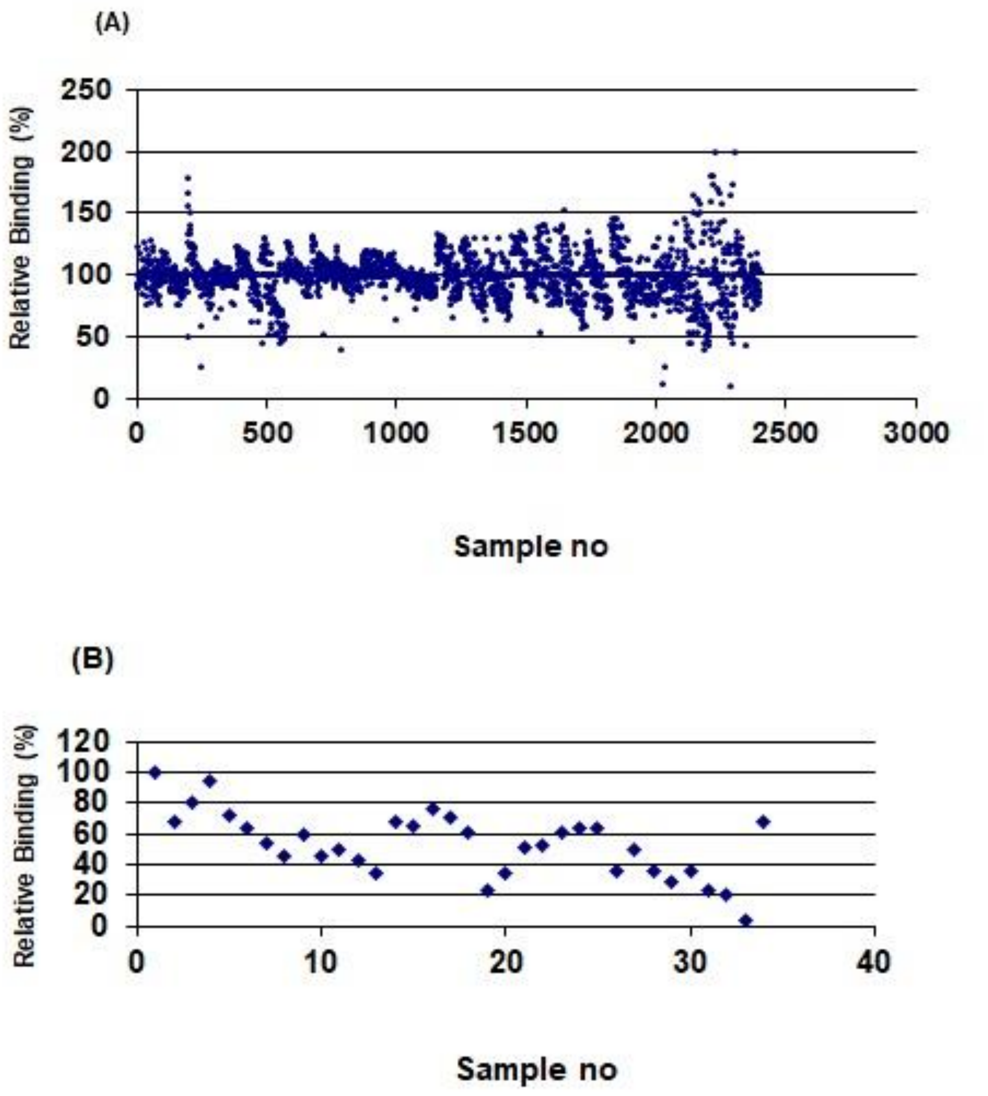

Supplementary Figure S1. Compound screening for inhibition of binding of S100A4 to nonmuscle myosin IIA (NMIIA) (A) Panel of 2,400 compounds were screened robotically and then **(B)** 33 of highly inhibitory hits that reduced S100A4 binding to immobilised fragment of NMIIA by 50% or more were further screened manually (Materials and Methods). The binding is shown relative to no compound addition set at 100% (Sample 1 in B), sample 33 is CT070909.

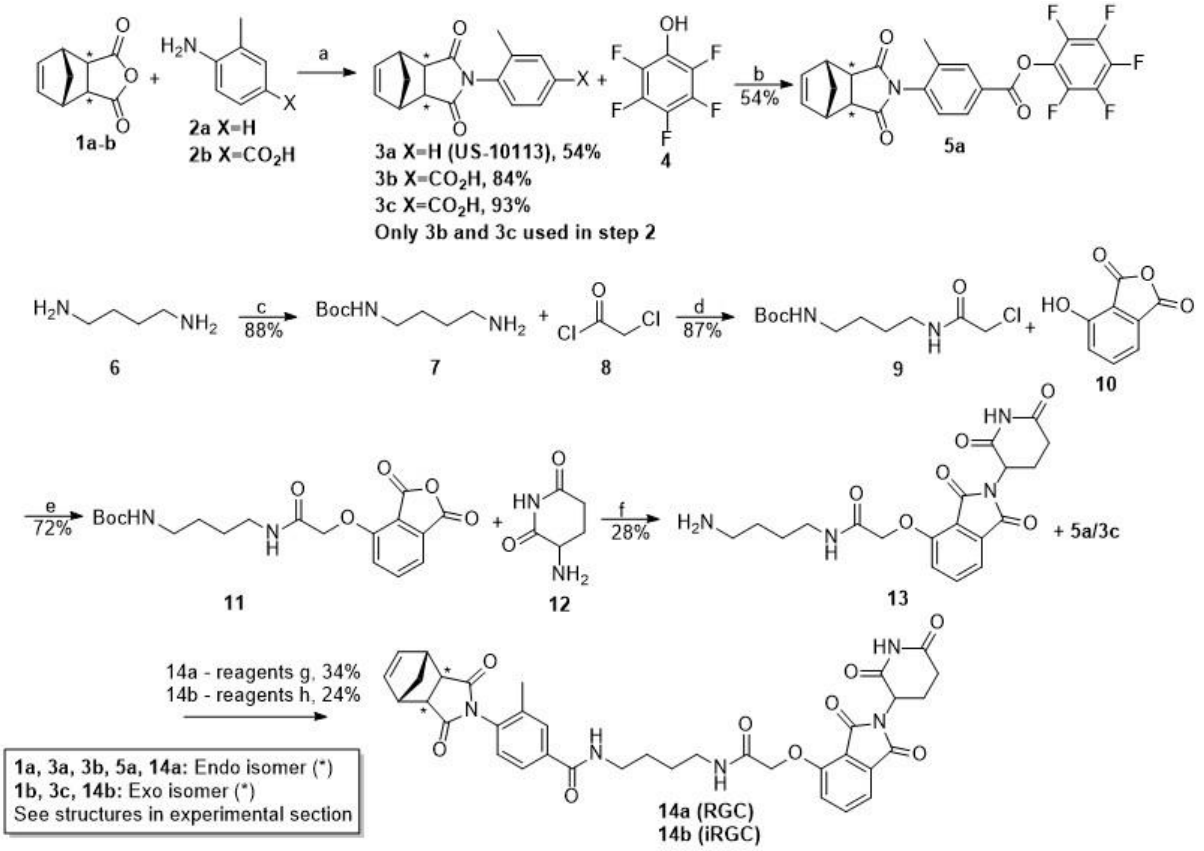

Supplementary Figure S2. Scheme for synthesis of US-10113, RGC and iRGC. Reagent and Conditions: (a) Acetic acid (AcOH), reflux, 16 hr; (b) 1-ethyl-3-(3-dimethylaminopropyl)carbodiimide hydrochloride (EDC.HCl), 4-dimethylaminopyridine (DMAP), dimethylformamide (DMF), room temperature (rt), 16 hr; (c) Di-tert-butyl dicarbonate (Boc_2O), dichloromethane (DCM), rt, 16 hr; (d) *N,N*-Diisopropylethylamine (DIPEA), tetrahydrofuran (THF), 0°C to rt, 16 hr; (e) Sodium hydride (NaH), THF, 0°C to reflux, 16 hr; (f) Potassium acetate (KOAc), AcOH, microwave (MW) 150°C, 12 mins; (g) DMAP, DMF, rt, 16 hr; (h) Hexafluorophosphate azabenzotriazole tetramethyl Uronium (HATU), DIPEA, DMF, 0°C to rt, 16hr.

39. Li, P.; Hwang, J.; Maier, J.M.; Zhao, C.; Kaborda, D.V.; Smith, D.; et al. Correlation between solid-state and solution conformational ratios in a series of n-(o-tolyl) succinimide molecular rotors. *Crystal Growth & Design* **2015,** *15,* 3561-64.

40. Fischer, E.S.; Böhm, K.; Lydeard, J.R.; Yang, H.; Standler, M.B.; Cavadini, S.; et al. Structure of the DDB1–CRBN E3 ubiquitin ligase in complex with thalidomide. *Nature (London)* **2014,** *512,* 49–53.

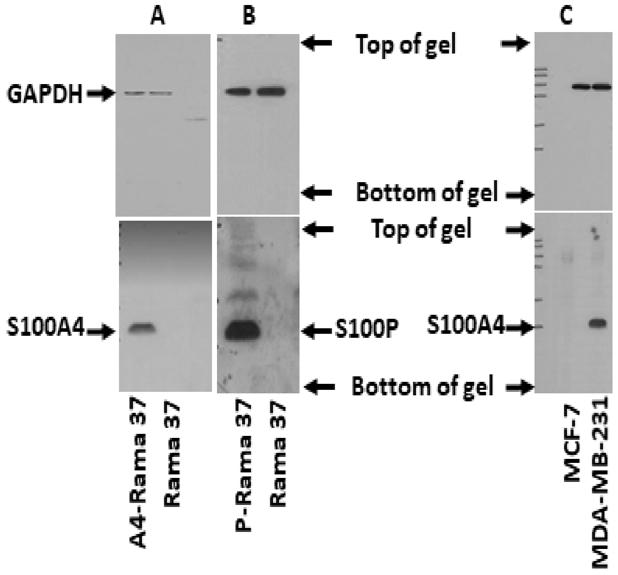

Supplementary Figure S3. Comparison of full-length Western blots for S100A4/P in rat and human breast tumour cell lines. (A) Metastatic S100A4-transfected Rama 37 (A4-Rama 37) and benign basal untransfected Rama 37 parental cells. **(B)** Metastatic S100P-transfected Rama 37 (P-Rama 37) and benign basal untransfected Rama 37 parental cells**. (C)** Less aggressive MCF-7 and more aggressive MDA-MB-231 breast cancer cells. Gels were loaded with an equal amount of 10 μg protein lysate, run and Western blotted as described in Materials and Methods. The top panels showed the control blots to confirm equal loadings using mouse monoclonal antibody to glyceraldehyde phosphate dehydrogenase (GAPDH) (Abcam, ab8245) followed by goat anti-mouse (Abcam, ab6708), and the bottom panels showed blots using antibodies to **(A)** S100A4, **(B)** S100P, and **(C)** S100A4 as described in Materials and Methods. Enhancer was used in all blots. The anti-S100P showed a small level of cross-reactivity with other proteins.

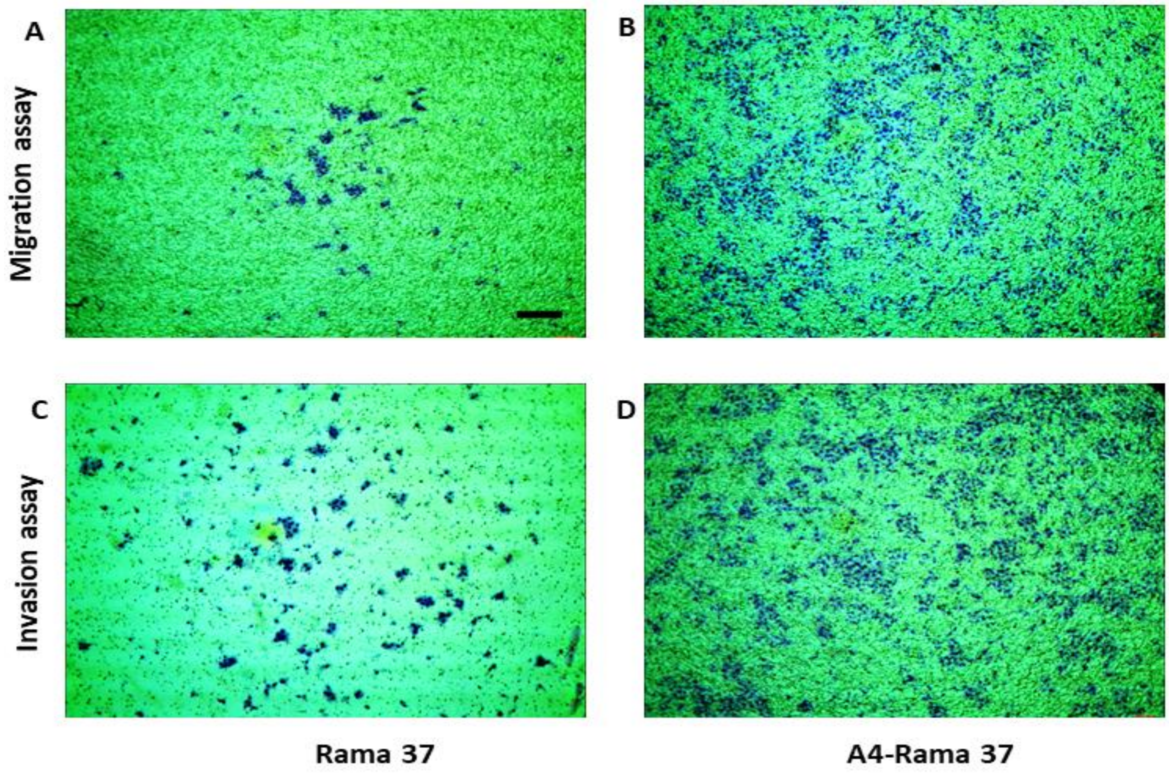

Supplementary Figure S4. Comparison of images of migration/invasion between benign Rama 37 and metastatic S100A4-transfected Rama 37 cells lines. Ten thousand cells of either **(A,C)** benign basal Rama 37 parental cells or **(B,D)** metastatic S100A4-transfected Rama 37 cells (A4-Rama 37) were added to Boyden chambers containing a semi-porous membrane with **(A,B)** no coating or **(C,D)** a coating of Matrigel and the chambers incubated for 24h as described in Materials and Methods/Supplementary Methods. Those cells migrating through the membrane were stained with a Diffquik histochemical kit and photographed in a microscope fitted with a Wratten 44 blue green filter. Original magnification x120. Scale bar = 200 μm.

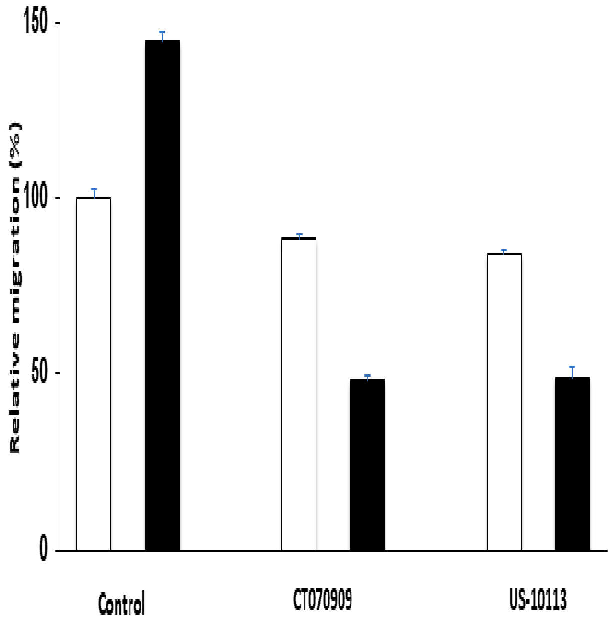

Supplementary Figure S5. Effect of CT070909 and its derivative US-10113 on the migration of rat breast cells. Chemicals or vehicle-alone controls (Control) at 33 µM were added to either parental Rama 37 (white columns) or S100A4-transfected Rama 37 (black columns) cell lines and assayed for cell migration as described in Materials and Methods. The number of cells migrating through Boyden chambers was recorded after 48h and the mean ± SE of 3 independent experiments is shown expressed as a percentage of the number migrating without any additions (% Relative migration).

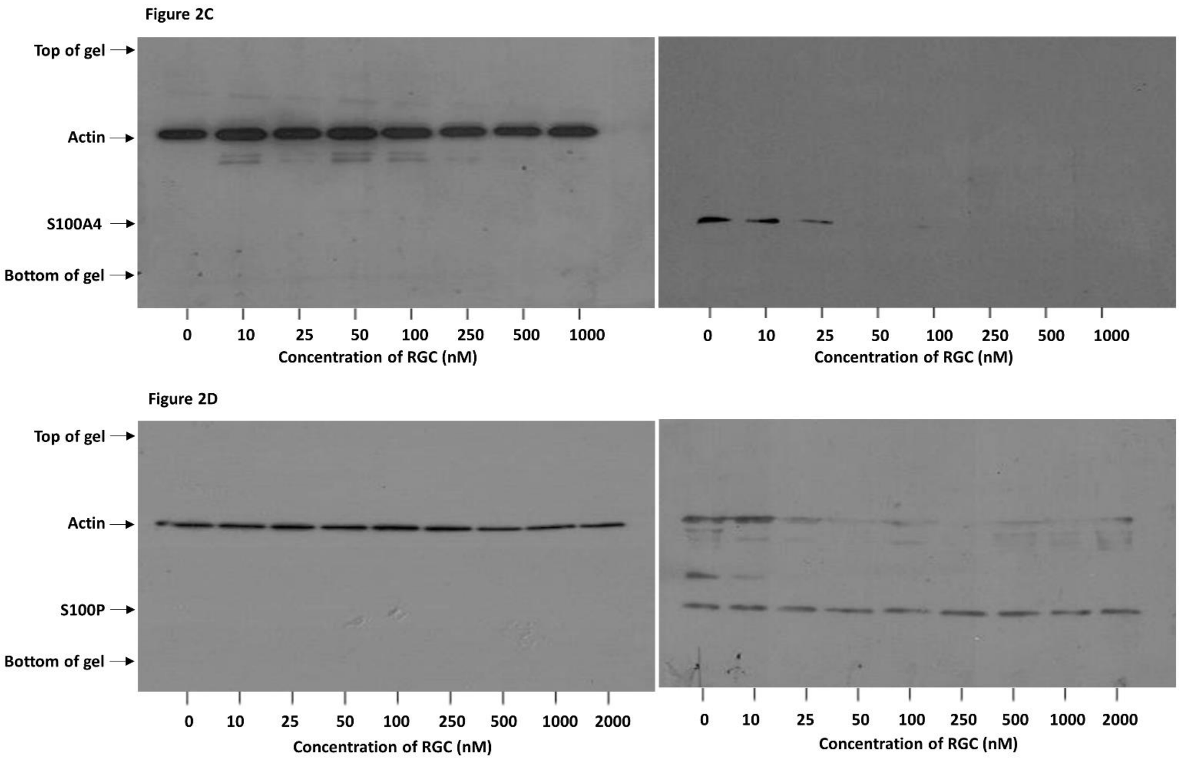

Supplementary Figure S6. Full length Western blots of effect of RGC on (Figure 2C) S100A4 in S100A4-transfected Rama 37 and (Figure 2D) S100P in S100P-transfected Rama 37 breast cancer cell lines. The lefthand panels show control blots for actin and the righthand panels blots for (Figure 2C) S100A4 and (Figure 2D) S100P. Enhancer was used in all blots except actin in Figure 2D (Materials and Methods). The anti-S100P showed a very small level of cross reactivity with other proteins.

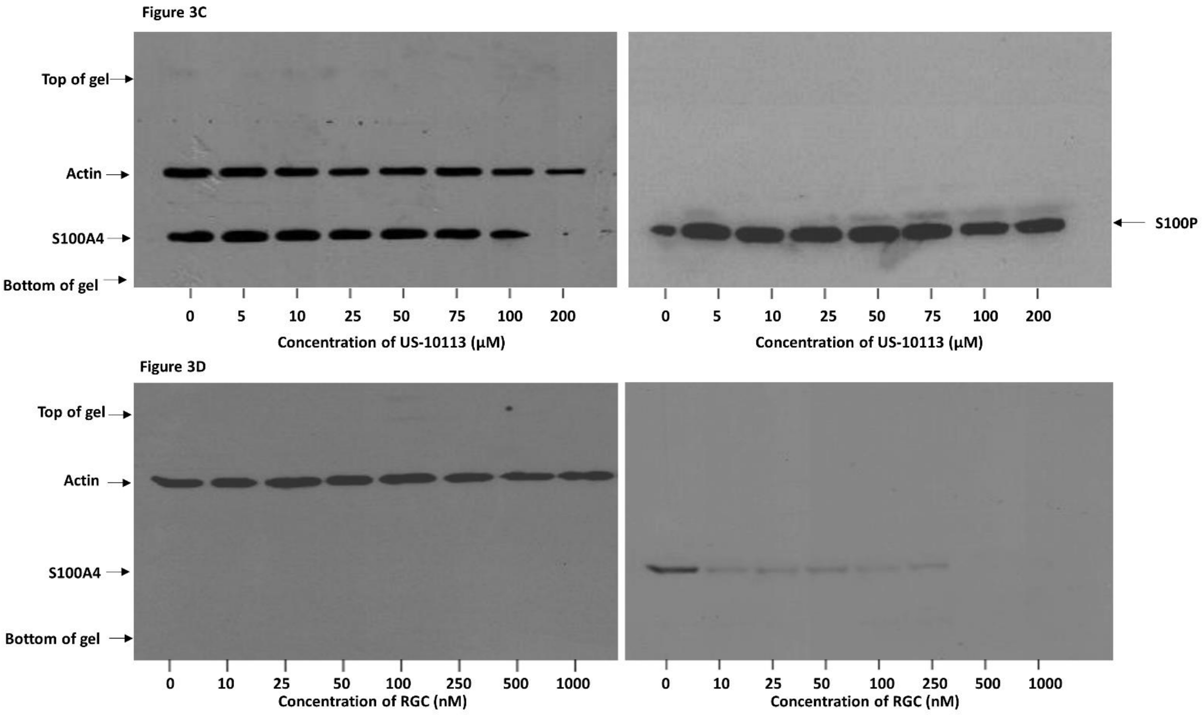

Supplementary Figure S7. Full length Western blots of effect of (Figure 3C) US-10113 and (Figure 3D) RGC on S100A4 or S100P in human MDA-MB-231 TNBC cell line. In Figure 3C lefthand panel, antibodies to both actin and S100A4 were included but in righthand panel antibody to only S100P was used. In Figure 3D lefthand panel, antibody to only actin and in righthand panel to only S100A4 was used.

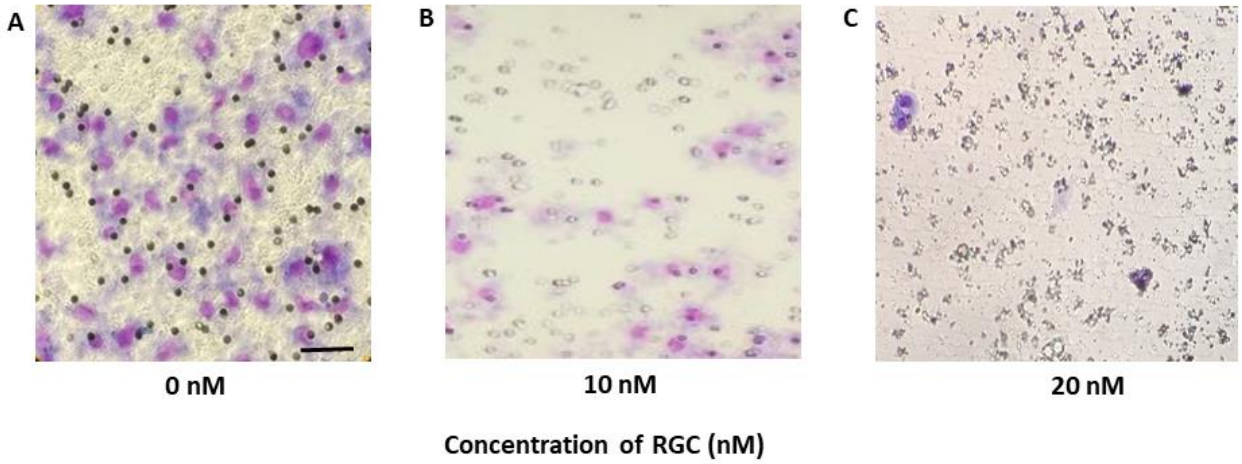

Supplementary Figure S8. Images of migration of TNBC MDA-MB-231 cell line with different concentrations of RGC. Six thousand cells of aggressive human TNBC MDA-MB-231 cells were added to Boyden chambers containing semi-permeable membranes with no coatings of Matrigel but with **(A)** 0 nM, **(B)** 10 nM or **(C)** 20 nM RGC and the chambers were incubated for 24h as described in Materials and Methods/Supplementary Methods. The cells migrating through the membrane were stained with a Diffquik histochemical kit and photographed in white light with no filter. Original magnification x160. Scale bar = 50 μm.

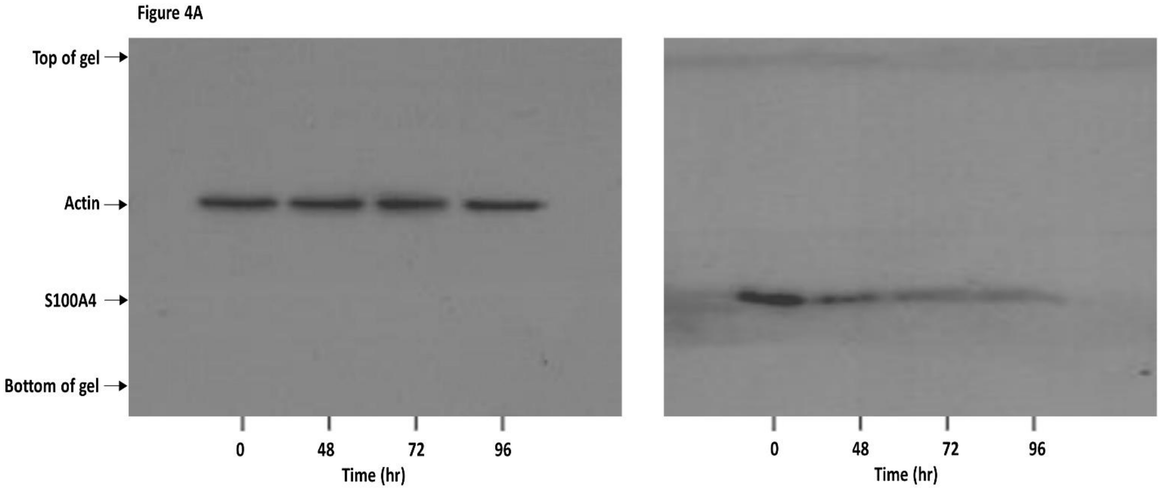

Supplementary Figure S9. Full length Western blots of Figure 4A for actin or S100A4 in MDA-MB-231 cells transiently transfected and depleted for S100A4 at different times after transfection (hr). Lefthand panel shows blot for actin and righthand panel blot for S100A4.

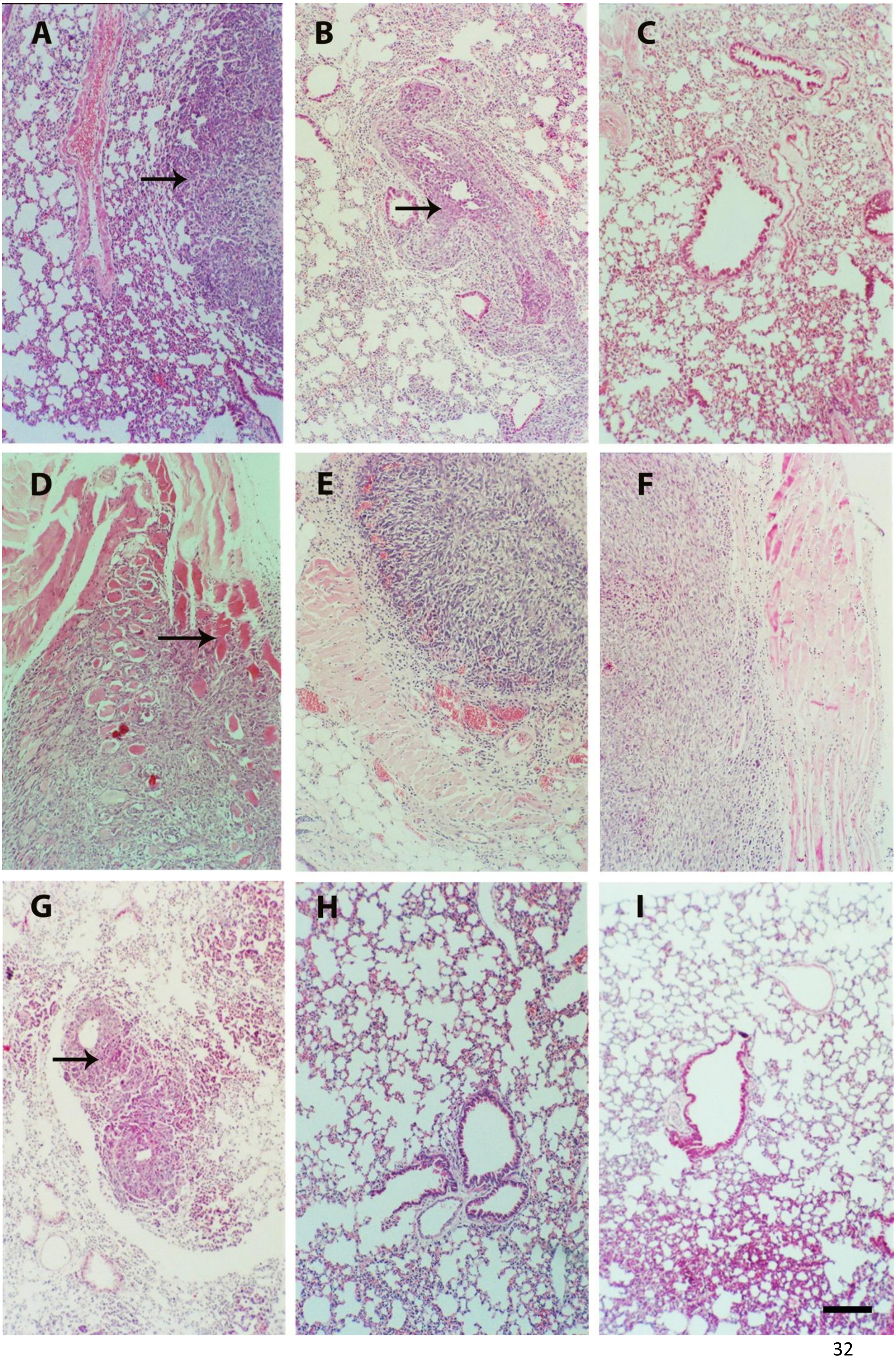
A
B
C
D
E
F
G
H
I

Supplementary Figure S10. Histology of mice bearing 4T1 tumours. Mice were injected **(A, B, C)** intravenously (iv) into the tail vein or **(D-I)** orthotopically (ortho) and subcutaneously (sc) into the mammary fat pad with syngeneic mouse 4T1 tumour cells and compounds were injected subcutaneously (sc) on the same day and every 2 to 3 days for 3 or 4 weeks, respectively (Materials and Methods). After this time autopsies were undertaken, and the histological sections of various tissues stained with haematoxylin and eosin (H&E) are shown (Materials and Methods) for different animal procedures. **(A, B, C)** Cells were injected iv with: **(A)** no further additions, **(B)** thalidomide (1.35 mg/kg), **(C)** RGC (0.07 mg/kg) or **(D-I)** ortho with: **(D, G)** no further additions, **(E, H)** RGC (0.007 mg/kg), **(F, I)** RGC (3.5 mg/kg) and the representative histologies of **(A-C, G-I)** lungs and **(D-F)** primary tumours adjacent to muscle are shown. Arrows indicate **(A, B, G)** metastases in the lungs or **(D)** invasion of the primary tumours through adjacent muscle. For 3.5 mg/kg some mice produced metastases similar to Fig.S12F. Statistical details are shown In Table 1. Original magnification x60. Scale bar=100 µm.

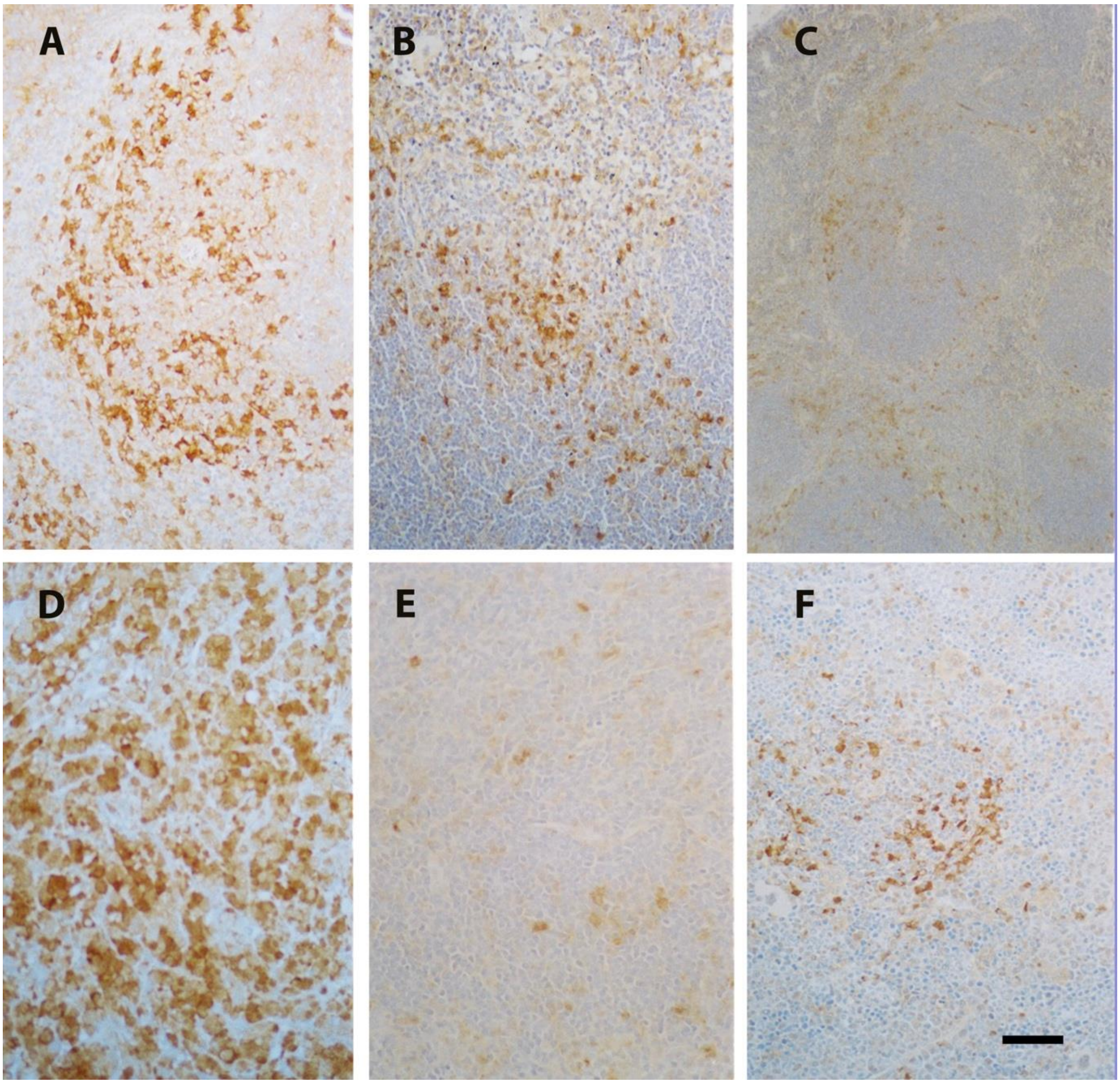

Supplementary Figure S11. Immunohistochemical staining of mouse spleens for S100A4. Mice were injected **(A, B, C)** intravenously (iv) or **(D, E, F)** orthotopically (ortho) with syngeneic mouse 4T1 cells and compounds injected sc on the same day and then every 2 to 3 days for 3 or 4 weeks, respectively (Materials and Methods). After autopsy, the splenic sections were immunohistochemically stained for S100A4 as described in Supplementary Methods, stained cells appear brown, and nuclei are counterstained blue. Representative stained sections are shown for iv injected cells for: **(A)** no compound, **(B)** thalidomide (1.35mg/kg), **(C)** RGC (0.7mg/kg) and for ortho injected cells for: **(D)** no compound, **(E)** RGC (0.007mg/kg), **(F)** RGC (3.5mg/kg). There were considerably less cells stained for S100A4 in **(C)** than in **(A, B)** and considerably less in **(E, F)** than in **(D).** Statistical details are shown in Table 2. Original magnification x154. Scale bar = 50 μm.

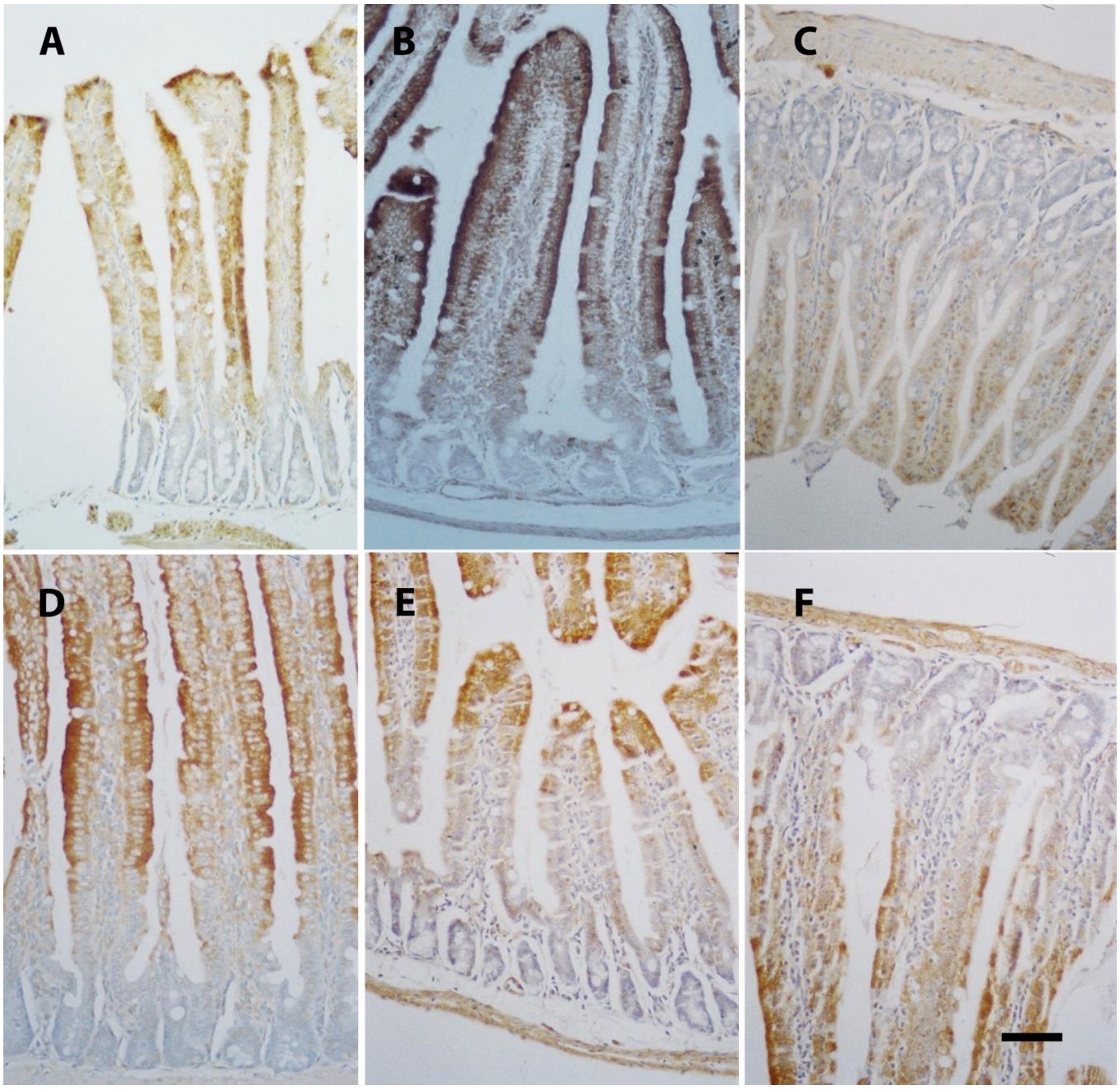

Supplementary Figure S12. Immunohistochemical staining of mouse colons for S100A4. Mice were injected **(A, B, C)** intravenously (iv) or **(D, E, F)** orthotopically (ortho) with syngeneic mouse 4T1 cells and compounds sc as described in Supplementary Figure S10 and Materials and Methods. After autopsy, colonic sections were immunohistochemically stained for S100A4 (Supplementary Methods), stained cells are brown and nuclei pale blue. Representative stained sections are shown for iv injected cells for: **(A)** no compound, **(B)** thalidomide (1.35 mg/kg), **(C)** RGC (0.7 mg/kg) and ortho injected cells for: **(D)** no compound, **(E)** RGC (0.007 mg/kg), **(F)** RGC (3.5 mg/kg). There were considerably less stained cells for S100A4 in **(C)** than in **(A, B)** and considerably less in **(E, F)** than in **(D)**. Statistical details are shown in Table 2. Original magnification x154. Scale bar= 50 µm.

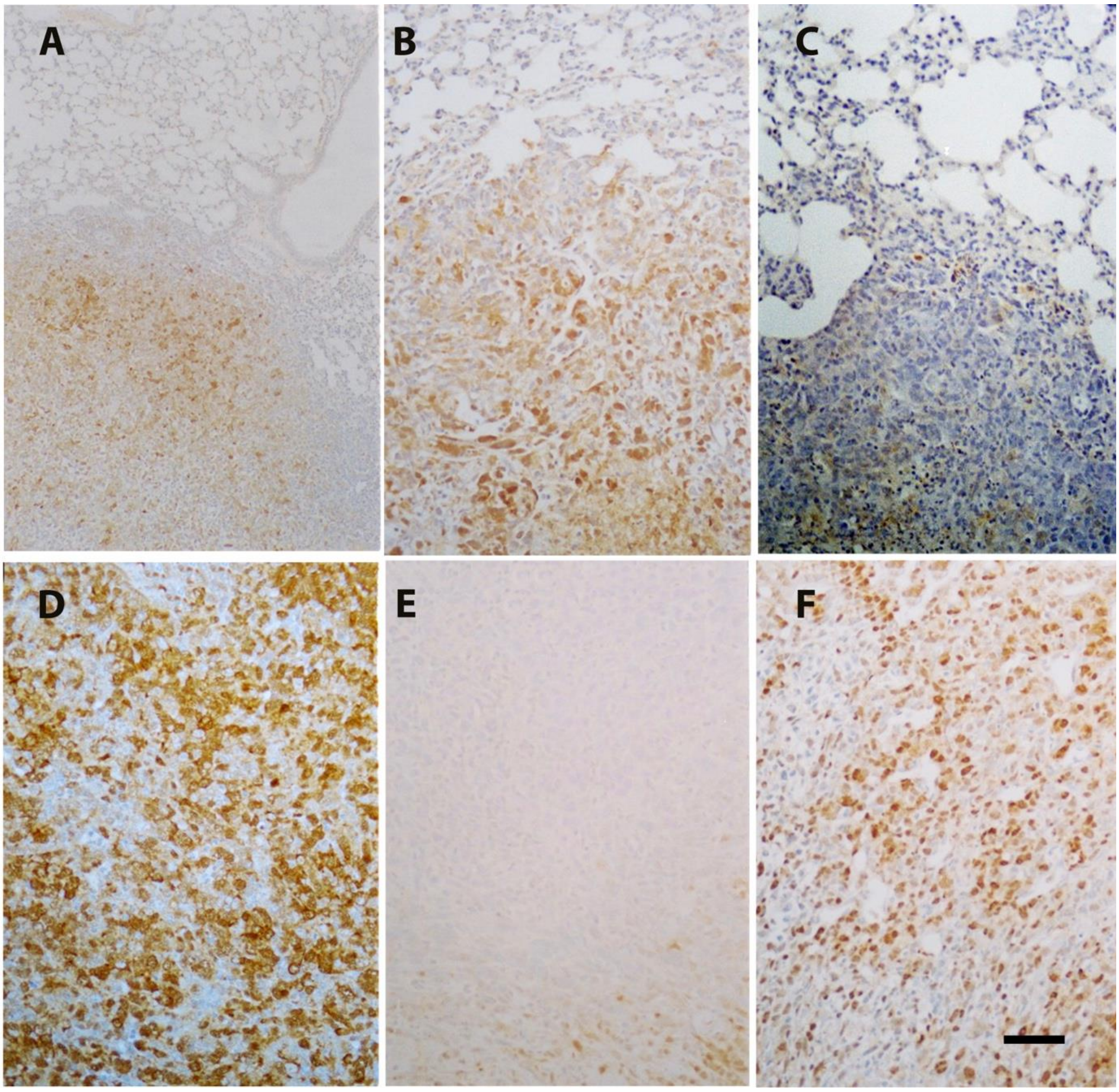

Supplementary Figure S13. Immunohistochemical staining of mouse tumours for S100A4. Mice were injected **(A, B, C)** intravenously (iv) or **(D, E, F)** orthotopically (ortho) with syngeneic mouse 4T1 cells and compounds sc as described in Figure S10 and Materials and Methods. After autopsy, histological sections of **(A, B, C)** lungs or **(D, E, F)** primary tumours were stained for S100A4 (Supplementary Methods), stained cells are brown, nuclei pale blue. Representative stained sections are shown for lungs for: **(A)** no compound, **(B)** thalidomide (1.35 mg/kg), **(C)** RGC (0.07 mg/kg) and for primary tumours for: **(D)** no compound, **(E)** RGC (0.007 mg/kg), **(F)** RGC (3.5 mg/kg). There were considerably less stained cells in **(C)** than in **(A, B)** and considerably less in **(E)** than in **(D)**, whilst **(F)** contained an intermediary level of stained cells. Statistical details are shown in Table 2. Original magnification x154. Scale bar=50 µm.

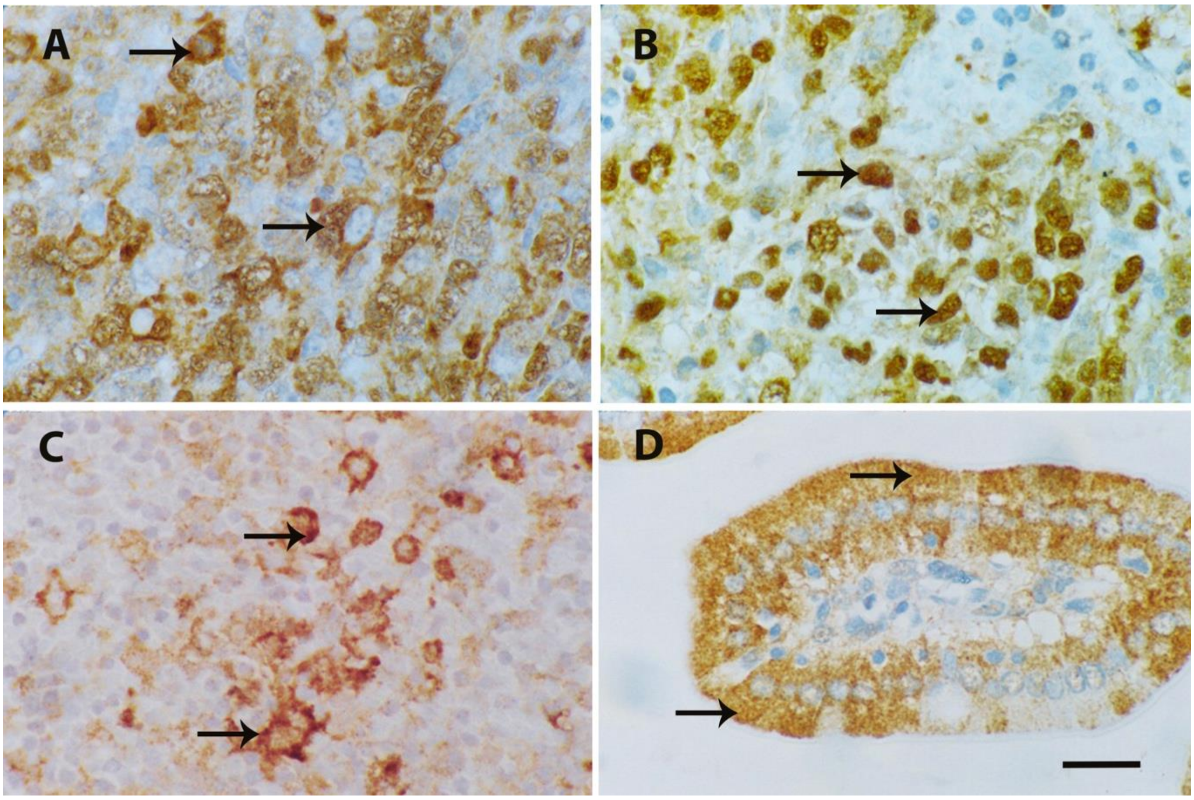

Supplementary Figure S14. Subcellular immunohistochemical staining for S100A4 in mouse tissues and tumours. Mice were injected orthotopically with syngeneic 4T1 cells and treated at the same time with RGC as described in Materials and Methods. After autopsy, sections from various tissues that were immunohistochemically stained for S100A4 (Supplementary Figures S11-S13, Table 2) were photographed at a higher magnification for: **(A)** primary tumour of mice exposed to no compounds, **(B)** primary tumour of mice treated with RGC (3.5 mg/kg), **(C).** Spleen of mice treated with RGC (3.5 mg/kg) and **(D)** colon of mice treated with RGC (3.5 mg/kg). Arrows show cell staining for S100A4 that was predominantly cytoplasmic in **(A)** became much more nuclear in **(B)** without corresponding changes in cellular location in **(C)** spleen or in **(D)** colon, these remained predominantly cytoplasmic. Statistical details are show in Table S6. Original magnification x480. Scale bar=20 µm.

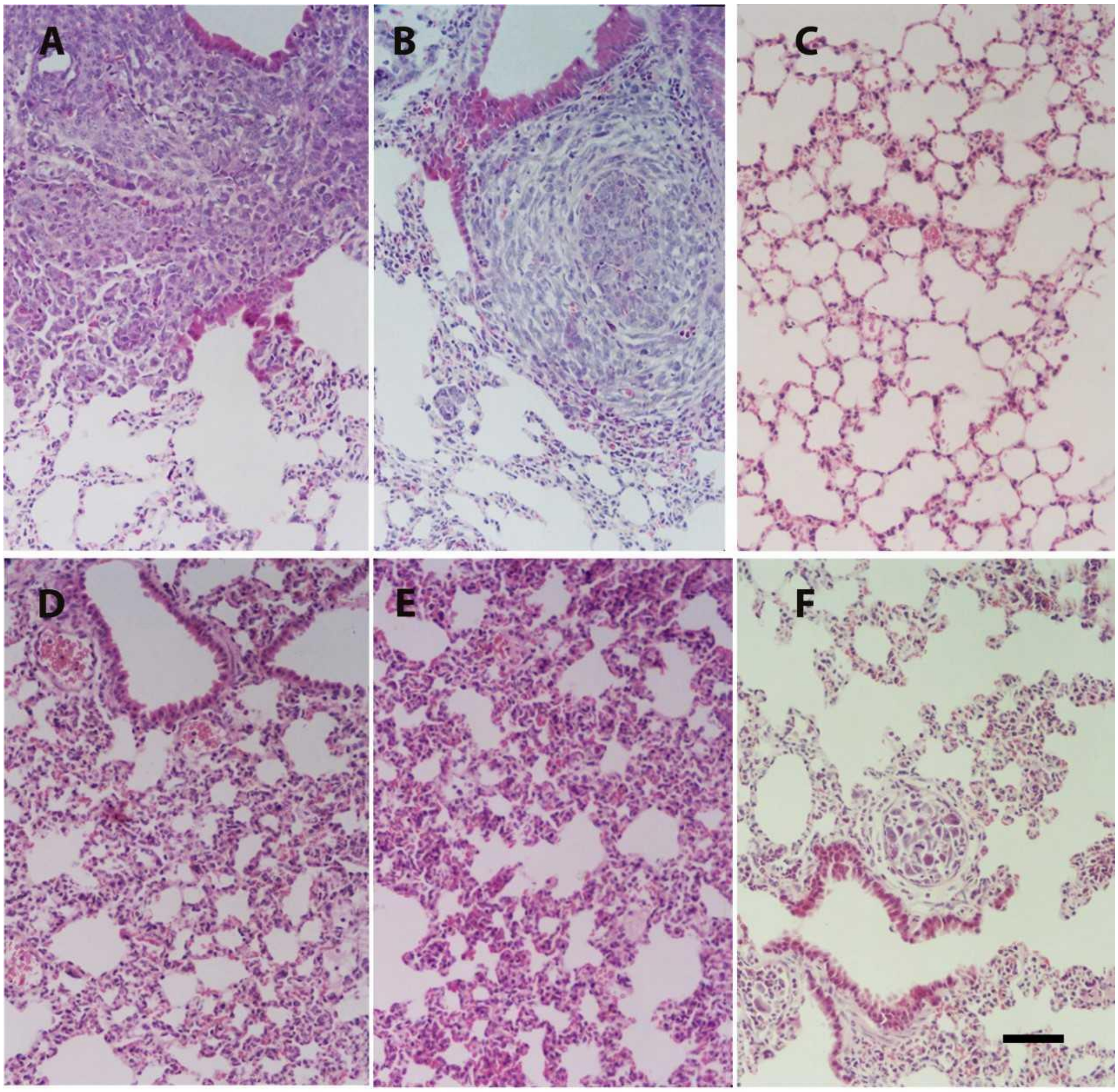

Supplementary Figure S15. Histology of mouse lung lesions after addition of compounds. Mice were injected intravenously (iv) with syngeneic mouse 4T1 cells and compounds were injected sc starting 14 days later as described in Table S7. After autopsy at 29 days, sections of lungs were stained with haematoxylin and eosin (H&E) (Materials and Methods). Compounds injected sc were: **(A)** none, **(B)** thalidomide (1.35 mg/kg), **(C)** RGC (0.07 mg/kg), **(D)** RGC (0.7 mg/kg), **(E, F)** RGC (3.5 mg/kg). Representative sections show that there were experimentally induced metastases present in lungs of mice in **(A, B)** but not in some mice in **(C, D)**, and the highest dose

of RGC produced mice **(E)** without and **(F)** with lung experimental metastases. Statistical details are shown in Table S7. Original magnification x154. Scale bar=5 μm.
